# Sleep and Dreams as Reflected by Science Fiction Literature and Films—Anything to Learn From?

**DOI:** 10.1111/jsr.70183

**Published:** 2025-08-21

**Authors:** Dieter Riemann, Christoph Nissen, Pierre A. Geoffroy, Bernd Feige, Jason Ellis

**Affiliations:** ^1^ Department of Psychiatry and Psychotherapy Freiburg University Medical Center, Medical Faculty Freiburg Germany; ^2^ Department of Psychiatry Geneva University Hospital Geneva Switzerland; ^3^ Département de psychiatrie et d'addictologie FAP‐HP, GHU Paris Nord, DMU Neurosciences, Hopital Bichat ‐ Claude Bernard Paris France; ^4^ Université Paris Cité, NeuroDiderot, Inserm Paris France; ^5^ Centre ChronoS, GHU Paris – Psychiatry & Neurosciences Paris France; ^6^ Department of Psychology Northumbria University Newcastle upon Tyne UK

**Keywords:** dreams, science fiction, sleep

## Abstract

Sleep and dreams are frequent themes in science fiction (Sci‐Fi) literature and films, often used to explore questions about consciousness, reality, technology and the human experience. Sci‐Fi authors and filmmakers utilise the enigmatic nature of sleep and dreams to blur the boundaries between reality and imagination, raising philosophical questions or extrapolating the effects of futuristic technologies on human life. In this article, we want to highlight some areas that have been recurring themes relating to sleep and dreams in Sci‐Fi. These will include the concepts of so‐called hypno‐paedagogics, space hibernation, brain machine interfaces, electrostimulation, genetic engineering and the impact of substances (viruses, bacteria, drugs, toxins) on sleep and dreams. We will then confront Sci‐Fi concepts with what is known from contemporary sleep science and judge what might be feasible, or not, in the future. A question we also want to address is how the relationship between sleep science and sleep Sci‐Fi can be conceptualised: whether novel concepts have been instigated by Sci‐Fi and taken up by sleep science or whether Sci‐Fi merely reflects state of the art topics of sleep science, with just adding a touch of fiction.

## 1 Introduction

The idea to write an article about the relationship between contemporary sleep science and science fiction (Sci‐Fi) literature and films arose when conceptualising this special issue of the Journal of Sleep Research (JSR) about the future of sleep medicine. It seemed promising to include a manuscript elucidating what Sci‐Fi can offer clinicians and scientists in the sleep field: does Sci‐Fi just ‘overstretch’ or extrapolate what is already known from the current state of the science of sleep medicine and research, or can we find novel, testable concepts there?

Thus, we will review Sci‐Fi literature and films: please note that it is not possible for us to arrive at a comprehensive review with a systematic literature search trying to identify (almost) everything ever published in Sci‐Fi literature and films. This would be far beyond our intention and would probably need engagement over a longer period and much more space than we have here. As such, we primarily include the most popular and well‐known literature and film from the Sci‐Fi area with the intention to ensure accessibility for the reader. These summaries are followed by brief descriptions of the state of the art of current sleep science and a conclusion framed by what is realistic or not in the future from our personal points of view.

To give this article a specific idiosyncratic Sci‐Fi touch, we decided to rely, in addition to our own writing capabilities, on the help of ChatGPT. We did so with a twinkle, utilising a technology which was considered Sci‐Fi until relatively recently.

## 2 Hypnopaedagogics

Hypnopaedagogics, also known as sleep‐learning or hypnopaedia, explores whether it is possible to acquire knowledge, skills, or behavioural changes by exposing the brain to information while asleep. The concept has fascinated both scientists and Sci‐Fi writers; though, from a present scientific point of view, it remains rather unsubstantiated (see below). Interestingly, the idea that the sleeping brain is able to consolidate and process information acquired prior to sleep has become one of the hot topics of sleep research (see below).

In general, Sci‐Fi depicts hypnopaedia as an advanced or dystopian form of education, where individuals are conditioned or programmed to learn new content—ranging from languages to societal rules—while asleep. It reflects themes of control, education and the manipulation of the mind in the future or from technologically advanced societies. Interestingly, the more general aspect of learning effortlessly (‘Nürnberger Trichter’, Nuremberg Funnel; Harsdörffer (1647): a method by which knowledge can be acquired without effort) while one is sleeping is not so much the focus of this literature, apart from one exception (see below—Robert Heinlein: *The Moon is a Harsh Mistress*, 1966). However, the concept has been extremely popularised and commercialised—there are numerous advertisements to find (mainly in the 70s and 80s, but also more recently: just google ‘learning during sleep’ and you will find lots of commercial offers to enhance your nocturnally acquired knowledge, but be careful with your credit card!) selling gadgets which promise new learning effortlessly ‘overnight’ just by listening to new vocabulary or any other new information. Needless to say, this does not work, at least not for complex learning processes!

Probably the earliest mention of this concept in Sci‐Fi literature was by Hugo Gernsback in 1925: *Ralph 124C 41+. A Romance of the Year 2660*. Gernsback described a new device called the ‘Hypno‐Bioscope’ which can transmit information, via a headband to sleeping people, enabling them to learn not only when awake but also when sleeping. It is interesting to note that Gernsback was not only a writer, but also an inventor and engineer, being involved in Radio and TV broadcasting. He was born in Luxemburg and died in New York and is credited to be one of the first Sci‐Fi writers. Probably the most famous example of hypnopaedia in Sci‐Fi is found in Aldous Huxley's *Brave New World* (1932). Hypnopaedia here is used as a method of acoustic indoctrination from an early age (even at the embryonal stage). Citizens are subjected to sleep‐learning to internalise social norms and reinforce the values of the ‘World State’, a totalitarian society where individuals are conditioned to accept their social roles without question. Hypnopaedia in this context does not teach specific subjects like mathematics or science but is highly effective for moral education and instilling unconscious biases, using monotonous slogans to ensure conformity. In *Brave New World*, hypnopaedia is used as a tool of control, with individuals conditioned to accept the status quo. Furthermore, by using sleep‐learning, the state diminishes the individuality and free will of its citizens, turning them into products of their conditioning rather than individual thinkers. Although not explicitly focused on hypnopaedia, Ray Bradbury's *Fahrenheit 451* (1953) includes elements of sleep‐learning through the use of constant media exposure. Characters are bombarded by information, especially through their ‘seashell’ ear radios that often play even while they are asleep. This creates a passive absorption of state‐approved messages, which mirrors the sleep‐learning concept of subtly shaping minds without active learning. The continuous flood of information, even during sleep, reflects how individuals in the novel are conditioned to conform to a society that values ignorance over knowledge. As noted earlier, in Robert Heinlein's *The Moon is a Harsh Mistress* (1966) sleep‐learning devices called ‘electromechanical educators’ are used to quickly teach skills to workers on the moon colony. These devices can implant knowledge, particularly practical skills, directly into the minds of sleeping or semi‐conscious individuals. This process saves time and bypasses traditional learning, showing a more practical and less dystopian version of hypnopaedia than in *Brave New World*. In this case, hypnopaedia serves as a tool for practical knowledge acquisition, focusing on the benefits of rapid learning in a highly functional society. Heinlein presents hypnopaedia as a positive technological innovation, reflecting how sleep‐learning could be harnessed for good rather than control.

In scientific reality, the concept of hypnopaedagogics has always been highly controversial, and scientific research on the topic has yielded, to put it mildly, no convincing evidence for the concept. Ataei et al. (2023) published a historical review on learning during sleep in humans with respect to the scientific literature. The authors were able to identify 51 research papers between the early 40s and today. A basic precondition for the concept of learning during sleep is that the brain is capable of processing information during sleep, even if the sleeper is unconscious. In summary, they suggest ‘…several studies support the notion that simpler forms of learning, such as habituation and conditioning, are possible during sleep’. With respect to more complex forms, like procedural or declarative learning (i.e., learning new vocabulary of a foreign language) the evidence is far more negative. Interestingly, when examining neural markers, it seems that some kind of processing of the applied information takes place, but on a behavioural level it remains inconclusive whether a transfer from sleeping to wake occurs. The only exception to this was found for studies investigating lucid dreaming during REM sleep. As such, the existing scientific evidence does not support far‐reaching claims of complex learning or conditioning processes taking place when applying new material during sleep. In their conclusion, Ataei et al. (2023) suggest more sophisticated methodological approaches are needed, in future studies, to homogenise and improve research in the area. As the scientific literature in this review starts in the 1940s, it appears that for hypnopaedia Sci‐Fi publications preceded or even instigated scientific effort.

Conclusion: The current state of the science suggests that in the future all of us will still have to acquire new material during conscious life, be it as a pupil, student, or scientist. It appears today that there is no easy, effortless alternative to consciously engaging with and memorising new important information. Interestingly, in contrast, there are manifold studies (for overview see: Brodt et al. 2023) demonstrating that substantial consolidation of material newly acquired prior to sleep is indeed taking place during sleep.

## 3 Space Hibernation (Cryosleep)

Space hibernation, alternatively termed cryosleep or hypersleep, is a popular concept in Sci‐Fi that allows humans to survive long‐duration space travel by slowing down or halting biological functions for extended periods of time. This concept arises from the challenge of interstellar travel, where distances between planets or stars can take years, decades, or even centuries to traverse, rendering normal sleep/wake schedules rather impractical for astronauts. Although purely fictional at present, space hibernation is a fascinating intersection of biology, technology and narrative, serving multiple purposes in Sci‐Fi stories: conserving resources, reducing psychological strain and addressing the immense time scales involved in space travel. Cryosleep or hypersleep refers to placing individuals in a state of suspended animation (SA) by dramatically slowing down their metabolic processes, reducing or halting biological aging and preserving the body for future revival. Cryosleep is often depicted as a state of immense vulnerability, where characters are dependent on technology to survive. This becomes a point of tension in many Sci‐Fi stories, as malfunctioning cryo‐systems or sabotage can lead to deadly outcomes—which may underscore the vulnerability of sleep itself. Another common theme in space hibernation stories is the idea of temporal dislocation. Characters who spend years or even centuries in cryosleep wake up in futures that are unfamiliar, adding emotional and philosophical depth to the narrative. This temporal gap can lead to feelings of alienation and the loss of connection to loved ones, societies, or even one's own identity. Some Sci‐Fi works take the concept of hibernation beyond artificial technology, imagining it as a biological adaptation or evolution to survive space. However, none of the Sci‐Fi works explain how hibernation or cryosleep would be induced and sustained in a scientifically testable manner.

Arthur C. Clarke's *2001: A Space Odyssey* (1968) is one of the most iconic examples for this genre, where astronauts are placed in cryosleep for long interstellar journeys. They remain in a vulnerable state, and in the novel, this vulnerability becomes a key plot point when HAL 9000, the AI aboard the ship, manipulates their cryosleep to control the mission. This book has been made even more iconic by its adaptation for film (Space Odyssee 2001, directed by Stanley Kubrick). In *The Ice People* (*La Nuit des Temps*, 1968) by René Barjavel, archaeologists uncover a couple who had been cryogenically frozen for thousands of years. The novel explores cryosleep not just as a survival mechanism, but as a metaphor for lost civilisations and the hope of renewal. In Dan Simmons' book *Hyperion Cantos* (1989), space travellers use cryosleep to cross vast distances in space. Simmons explores the psychological and physical effects of prolonged hibernation, raising questions about identity and disorientation when the characters wake up in different times or settings. In Alastair Reynolds' *Revelation Space* series (2000–2007), the concept of human beings enduring long periods of time in cryosleep is depicted, including the psychological and biological impacts of such hibernation. Here, hibernation is not only necessary for space travel but also becomes part of the human experience of traversing massive periods of time, especially when combined with near‐light‐speed travel. In Hugh Howey's *Wool trilogy* (2011–2013), cryosleep becomes a pivotal narrative device linked to societal control and long‐term survival. Characters are placed in cryogenic suspension as part of a covert plan to outlast a global catastrophe. The ethical implications of forced cryosleep, memory manipulation and selective awakening add a dystopian layer to the technology's use, emphasising how hibernation can be repurposed not for exploration, but for domination. In James S.A. Corey's *The Expanse* series (2011–2021), characters use cryosleep for long journeys. The series addresses the medical and psychological risks associated with entering such a state, including the loss of muscle mass and the mental toll it takes on travellers who wake up disoriented after long periods of unconsciousness. Kim Stanley Robinson's *Aurora* (2015) also explores hibernation aboard a generation ship. While most passengers are awake, certain key individuals enter hibernation to ensure they are available at critical stages of the journey. Robinson's work presents hibernation as a strategy for managing limited resources and preserving specific human expertise over a long mission.

The concept of space hibernation also features prominently in film: Ridley Scott's Alien (1979) and its sequels heavily rely on cryosleep. In the opening scenes, the crew of the Nostromo is awakened from hibernation upon reaching a new planet. Cryosleep allows the crew to save resources (food, water, oxygen) during their journey while emphasising their isolation and vulnerability in space. Interestingly, in the final scene in Alien, Ripley is seen ‘sleeping peacefully’ in the cryochamber after defeating the xenomorph, perhaps as a passive nod to the recuperative nature of sleep. Sunshine (2007) features a crew placed in cryosleep for part of their journey toward the sun. The film touches on the disorienting effects of coming out of hibernation and how the psychological stress of space travel impacts decision‐making and personal relationships. Pandorum (2009) explores space hibernation gone wrong. The crew members wake up from hypersleep on a ship adrift in space, suffering from memory loss and gradually discovering the nightmarish consequences of their prolonged stasis. The film taps into the psychological and physical horror of an extended brain state, where memories and mental states become unstable. Avatar (2009) shows humans travelling in cryosleep to the distant moon Pandora. The film emphasises the cultural and emotional consequences of waking up after a journey that spans light years, where the passage of time leaves behind loved ones and familiar worlds, creating a sense of profound separation. In Christopher Nolan's Interstellar (2014), hypersleep is used to preserve astronauts' physical health during long‐distance space travel. The film explores the emotional toll and sacrifice of spending decades in hibernation, knowing that significant time is passing for their loved ones on Earth. Passengers (2016) uses hibernation as the core plot device. In the film, thousands of passengers are put into hibernation aboard a ship travelling to a distant planet. The story revolves around one passenger waking up 90 years too early, which forces the character to grapple with the loneliness and existential questions of being stranded between worlds.

While space hibernation remains fictional, recent advances in science at least hint at the possibility of inducing hibernation‐like states in humans, particularly for medical purposes. In nature, certain animals (such as bears, squirrels and some species of birds) enter states of torpor or hibernation to survive extreme conditions—an observation that has undoubtedly inspired authors. During hibernation, their metabolic rates drop dramatically, conserving energy and reducing the need for food and oxygen. These natural processes are under investigation to explore whether human biology can be modified to mimic these states for long‐term space travel. Researchers have induced hypothermia in medical settings to slow metabolism during surgery, hinting that similar methods might one day be used to induce hibernation for astronauts (for a general overview see, e.g., Ohba and Yamaguchi 2025; Liu et al. 2024; Vrij et al. 2023). Of note, cryonics (the process of preserving life, or ‘pausing death’, through subfreezing) is something that is commercially available today.

Both NASA and the European Space Agency (ESA) have explored the potential for human hibernation as a means of addressing the challenges of long‐duration space missions, such as those to Mars or beyond (see, e.g., Regan et al. 1985; Feys et al. 2024). Torpor (a state of deep sleep or reduced metabolic activity) is being investigated as a way to reduce resource consumption (like food and oxygen) and the psychological stress of long isolation while being awake. The idea would be to place astronauts into a state of ‘synthetic torpor’ using drugs or medical devices to lower body temperature, reduce metabolism, and possibly prevent bone and muscle loss. Inducing a hibernation‐like state in humans would involve further significant challenges, such as preventing tissue damage, managing nutrient supply and avoiding the potential psychological effects of long‐term unconsciousness. Researchers are working on understanding how to safely bring someone out of hibernation without any major adverse effects.

### 3.1 Conclusion

Space hibernation is a staple of science fiction, providing a solution to the challenges of interstellar travel. Whether it is portrayed as cryosleep in *Alien* and *Interstellar* or as biological adaptation in *Revelation Space*, hibernation in sci‐fi taps into profound questions about time, identity and the future of humanity in space. While real‐world science has not yet achieved this (see Liu et al. 2024: ‘At present, artificial hibernation technology is not mature, and research faces various challenges’.), ongoing research into torpor and metabolic control suggests that at least elements of this dream could one day become a reality for medical purposes.

## 4 Brain Machine Interface(s) and Electrosleep

Brain‐machine interfaces (BMIs), also known as Brain‐computer interfaces (BCIs), are systems that enable direct communication between the brain and an external device. Although the term ‘interface’ suggests bidirectionality, the most common application is understood to be from brain to device, bypassing the traditional pathways of movement and speech, allowing one to effect the outer world by ‘thoughts’. While BMIs have been developed for a variety of purposes—such as assisting people with Parkinson's disease (deep brain stimulation), paralysis, controlling prosthetic limbs, or interfacing with virtual environments (see, e.g., Kinfe et al. 2025)—research is also expanding into the realm of sleep. Bidirectional BMIs would function by measuring electrical activity in subjects and by applying electrical currents to them—as such, the topics of BMIs and electrostimulation belong in one chapter. As we will see, electrostimulation methods range in a continuum from influencing the subjects' general brain state to eliciting concrete perceptions, leading to a relatively broad BMI concept.

In Sci‐Fi, BMIs have often been depicted in futuristic societies where human minds are seamlessly integrated with technology. The integration of sleep and BMIs opens the door to a range of speculative possibilities, from observation of dreams in real‐time and potentially their manipulation to memory extraction and even virtual reality worlds accessible during sleep. Interestingly, probably the first mention of a BMI, though not explicitly, comes from a classic gothic novel, *Frankenstein* by Mary Shelley (1818). In the book, it is never directly described how Viktor Frankenstein revives his creature, which was stitched together from dead body parts, but the iconic film (Frankenstein 1931) with Boris Karloff as the creature is more dramatic and ascribes a central role in the ‘awakening’ procedure to electricity. Thus, at least in the film, electricity is considered a major force which even can wake up the dead, qualifying the depicted apparatus in the film as a BMI in its broadest sense. Electrostimulation—the application of electrical currents to stimulate or modulate the nervous system—has emerged as an exciting area in sleep science (see Krone et al. 2025) and is frequently explored in Sci‐Fi. This technique involves using electrical impulses to influence brain activity, which might be used to enhance sleep, induce specific sleep stages, or even manipulate dreaming. The possibilities it suggests, both in real‐world science and speculative fiction, include altering the human need for sleep, improving cognitive function and accessing consciousness in new ways.

Many books focus on BMI and electrosleep: *Neuromancer* (1984): William Gibson's novel, a foundational work of cyberpunk, imagines a world where people interface directly with the digital world through their minds. BMIs could similarly enable users to access virtual worlds while asleep, using their dream states to navigate immersive virtual environments, where time and space function differently. In *Altered Carbon* (2002), a novel by Richard K. Morgan (and later a Netflix series), the concept of transferring consciousness via technology plays a major role. Though it focuses more on the concept of ‘stacks’ (digital consciousness storage), it touches on the idea of suspending physical bodies and manipulating consciousness—concepts that could extend to sleep in future depictions of BMIs. In these series, technology is so advanced that people can be put to sleep or awakened instantly, which constitutes a step toward controlling not just sleep but consciousness itself.

The anime film Paprika (2006) explores the concept of using technology to enter and manipulate people's dreams. A device called the ‘DC Mini’ allows therapists to view and interact with patients' subconscious minds during their dreams. The film highlights both the therapeutic possibilities of dream manipulation and the potential ethical dangers of invasive technology. Christopher Nolan's film Inception (2010) explores the idea of entering and manipulating dreams through shared lucid dreaming technology. The characters use devices that create interconnected dream worlds, where people can influence or extract information from others' dreams. While the technical means through which this is done are not elaborated, the concept of controlling dreams through technology and entering shared dream spaces aligns with the potential of BMIs in manipulating or guiding dream content.

Probably one of the first mentions of electrosleep in the Sci‐Fi literature is the dissertation ‘Studien über den elektrischen Schlaf’ (studies on electrosleep) published by Oskar Roos in his medical dissertation in 1916. He tried to induce sleep in rabbits and dogs by applying intermittent direct electrical currents. Having only observational data to judge (the EEG—electroencephalography, was only discovered approximately 10 years later) he concluded that with his procedure of stimulating the cranium the experimental animals fell into a sleep‐like immobile state often lasting up to 1 h.

From a present point of view, it is doubtful whether really a sleep state was induced or whether the animals were just narcotised. The discovery of the EEG by Hans Berger in 1929 laid the scientific basis for BMIs and electrosleep: It demonstrated that electrical brain activity, as measured with electrodes attached to the scalp, is actually related to mental processes. This made it not only possible to measure certain brain states and mental processes but also opened the door to brain stimulation techniques.

BMIs and electrosleep depend on real‐time brain monitoring through EEG or other neural recording techniques. EEG is the gold standard for tracking the brain's electrical activity during different sleep stages. Traditional sleep studies often require participants to wear uncomfortable sensors or spend nights in labs for polysomnography. Significant progress has been made in the last decades concerning at‐home monitoring with simplified electrode sets and ‘wearables’ making it possible to monitor sleep less obtrusively, making it more likely that sleep research and sleep medicine in the future might leave the stationary sleep laboratory (see De Zambotti et al. 2024). This will likely enable more accurate diagnoses of sleep disorders, such as insomnia, sleep apnoea, or narcolepsy. Moreover, researchers will be able to monitor sleep stages over long periods, collecting large amounts of data to understand how environmental factors, stress, or daily habits affect sleep. This unobtrusive monitoring also is a precondition for practical BMIs.

BMIs and electrosleep approaches are being speculated about concerning sleep regulation, deep (slow wave sleep) sleep enhancement, treating sleep disorders, enhancing memory consolidation, targeted memory reactivation (TMR), brain plasticity, lucid dreaming and more general dream content.

In real‐world research, no BMI for sleep in its original sense exists at present. However, transcranial magnetic stimulation (TMS), and transcranial electrical stimulation (tES), particularly transcranial direct current stimulation (tDCS) and transcranial alternating current stimulation (tACS), have been studied for their effects on sleep (see Krone et al. 2025). These techniques involve applying either magnetic fields or weak electrical currents to the scalp to modulate brain activity in a targeted way, often with the goal of improving sleep quality or enhancing specific brain functions during sleep. The results up to now are not overwhelming, as Krone et al. (2025) state ‘…intense research efforts to use TMS or tES for sleep modulation have not yet delivered evidence based NIBS, Non Invasive Brain Stimulation, treatments in sleep medicine’. This may be due to unresolved methodological issues. More promise is seen in other approaches like closed‐loop stimulation, with the aim to specifically target certain waveforms to promote associated sleep functions, that is, those associated with memory consolidation. Thus, to quote Krone et al. (2025) again ‘Effective treatments in the area of sleep medicine seem in reach but require rigorously designed clinical trials to identify which NIBS strategies bring real benefit…’.

Conclusion: The convergence of BMIs and sleep research is a cutting‐edge area of neuroscience with vast potential (see, e.g., in its most extreme form: Musk 2019). From enhancing sleep quality and memory consolidation to exploring dream manipulation and cognitive health, BMIs could transform how we interact with our own minds during sleep. Both in real‐world research and in the imaginations of science fiction writers, the possibilities seem vast, offering exciting and sometimes unsettling visions of the future. Admittedly, at present, the practical realisation is not in near sight. Furthermore, immense ethical and societal issues would be coupled with the realisation of these possibilities. Who would control these technologies? Would that be within the realm of general healthcare or be just a lifestyle technology available for those who can afford it? Nevertheless, concerning future realisation, we do see a great potential for BMIs and electrostimulation in a more realistic way compared to other chapters of this article. That said, we strongly argue for strict ethical supervision and control in this field.

## 5 Genetic Engineering

Genetic engineering is the manipulation of an organism's DNA to alter its traits, and when applied to sleep, it opens up a fascinating and potentially transformative field of research. The genetics of sleep are complex, as sleep is regulated by a variety of genes that influence circadian rhythms, sleep cycles and individual sleep needs. By understanding and manipulating these genes, scientists aim to be able to better control and enhance sleep, addressing sleep disorders, improving cognitive performance, and even extending the amount of time people can stay awake without negative consequences. Genetic engineering of sleep has long been a topic of interest in SciFi, where it is often portrayed as a means to enhance human performance or create dystopian outcomes.

In novels like *Beggars in Spain* (1993) by Nancy Kress, genetically engineered humans, known as ‘Sleepless’, are created to no longer require sleep. These people are smarter, more productive and more successful than ordinary humans, leading to societal divisions and conflict between the *Sleepless* and the rest of the population. In the *Altered Carbon* universe by Richard K. Morgan, sleep is sometimes bypassed entirely in favour of advanced technologies that allow for sleeplessness, although at the cost of mental and physical side effects. Films like Gattaca (1997) explore the ethical and social ramifications of genetic engineering, though the focus is on broader genetic enhancement rather than sleep specifically. Still, the concept of a society stratified by genetic modifications applies to the potential future of sleep enhancement, where genetically engineered individuals could have significant advantages over those who remain ‘natural’.

Many genes are involved in regulating sleep patterns, sleep duration, circadian rhythms and sleep disorders (see for an overview: Kocevska et al. 2021; Yook et al. 2021). Advances in genomics and gene editing technologies like CRISPR provide new tools to explore the relationship between genes and sleep. Circadian rhythms are controlled by ‘clock genes’ like PER (Period), CRY (Cryptochrome), CLOCK and BMAL1, which work together to maintain the body's internal clock (for overview see: Shafer 2025). Mutations in these genes can lead to circadian rhythm disorders, such as delayed sleep phase syndrome (DSPS) or advanced sleep phase syndrome (ASPS), where individuals have difficulty falling asleep or staying awake at socially acceptable times. Variants in the PER3 gene have been linked to differences in sleep length. People with certain mutations in PER3 may need less sleep, while others require more. A mutation in the DEC2 gene has been identified in people who naturally sleep less than 6 h a night without apparent negative health consequences. This gene has drawn attention for its potential in creating genetically modified humans who need less sleep.

Several sleep disorders are linked to genetics: Narcolepsy, which is characterised by excessive daytime sleepiness, cataplexy and sudden sleep attacks, has been associated with variations in the HLA (Human Leukocyte Antigen) gene complex, which influences immune system function (see, e.g., Freeman et al. 2025). Insomnia also may have a genetic component. Large‐scale genetic studies have identified several genes, such as MEIS1, that increase the risk of insomnia (for an overview see Palagini et al. 2023). Similar results have been obtained for Restless Legs Syndrome (Schormaier et al. 2017). The structure of sleep, including the balance of REM (rapid eye movement) sleep and non‐REM sleep, is also genetically influenced. Studies involving twin populations have shown that about 40%–50% of sleep traits, such as sleep duration, are heritable (Madrid‐Valero et al. 2020).

Several research issues are presently debated. Can we use genetic engineering to reduce the amount of sleep that humans need at no cost to cognitive performance and health? This might be useful in professions that demand long hours, such as healthcare. In a future scenario, genetic engineering could allow humans to function optimally on just 4–5 h of sleep per night. This could enhance productivity and free up more time for daily activities. Can we make use of genetic manipulations to improve poor sleep in afflicted individuals or in any kind of sleep disorder? People with sleep apnea or insomnia could benefit from genetic modifications that correct the underlying genetic dysfunctions responsible for their condition. CRISPR technology could be used to repair mutations in genes linked to circadian rhythm disorders, effectively resetting a person's internal clock. Narcolepsy, which is often linked to the loss of hypocretin‐producing neurons in the brain, could be treated by using genetic engineering to restore these neurons or regulate hypocretin production. Can we target genes involved in sleep architecture, such as those regulating REM and slow wave sleep (SWS)? This could serve to develop novel interventions to improve sleep quality and enhance the brain's ability to consolidate memories and rejuvenate the body. Can we target aging‐related deteriorations of sleep quality and structure? Genetic engineering could help maintain youthful sleep patterns, promoting better cognitive function and physical health in older adults. Research into genes that regulate sleep in aging populations could lead to therapies that slow down the deterioration of sleep quality with age.

Conclusion: While much of the application of genetic engineering to sleep remains speculative, current research is actively exploring the genetic basis of sleep. Large‐scale genome‐wide association studies (GWAS) are aiming to identify genes linked to sleep duration, sleep quality and sleep disorders, offering potential targets for future genetic interventions. A major restriction may be the fact that many human behaviours are probably due to the influence of a variety of genes or indeed interactions between genes and the environment (epigenetics) and any intervention on a single gene may have unforeseen consequences in a network of genes and their related behaviours. Advances in CRISPR and gene therapy may pave the way for more precise manipulation of the genes involved in sleep regulation. The role of sleep genes is also studied in other species, such as flies or mice, to gain insight into how sleep duration and architecture can vary dramatically across the animal kingdom (see, e.g., Belfer et al. 2018).

This may offer clues for how genetic manipulation could influence human sleep. However, it must be critically examined whether these insights into highly genetically manipulated animal organisms might bear any relevance for humans. So, at present, the intersection of genetic engineering and sleep represents a promising but complex frontier in both science and speculative fiction. At present, we are not aware of any successful experimental genetic manipulation in humans impacting on sleep or sleep disorders. However, if once realised, these advancements would entail profound ethical and societal conflicts that would have to be carefully considered.

## 6 Substances and Sleep

The interplay between viruses, bacteria, drugs and toxins in relation to sleep has been a rich theme in both scientific inquiry and science fiction literature and film. These elements can influence sleep patterns, sleep quality and overall health, and in speculative narratives, they often serve as vehicles for exploring dystopian futures, ethical dilemmas and the fragility of human biology. Science fiction often explores the implications of viruses, bacteria, drugs and toxins on sleep, using them as catalysts for plot developments and thematic exploration. In the following paragraphs, we discuss some common themes and examples from literature and film.

In *Brave New World* (1932) by Aldous Huxley, citizens are pacified by a euphoric drug called soma, which provides a sense of euphoria and tranquillity, effectively managing their mental state and sleep without them even realising. The novel anticipates how pharmacology could govern not only behaviour but consciousness itself. In *Ashes* (1943), by René Barjavel, a future France collapses after technological failure. A subtle toxic atmosphere impacts physical and mental states, hinting at environmental sabotage of sleep and cognition. In *Dune* (1965) by Frank Herbert, the ‘spice’ melange not only enables interstellar travel and political control, but also induces altered states of awareness that blur the boundaries between sleep, prophecy and waking consciousness. In *The Andromeda Strain* (1969) by Michael Crichton, a deadly extraterrestrial microorganism threatens life on Earth, causing profound biological disruptions, including those affecting neural and sleep functions, while humanity scrambles to decode its lethal mechanisms.

Cinematic and TV Sci‐Fi also explores how substances, natural or engineered, can manipulate sleep, dreams and consciousness. In The Crazies (2010), directed by Breck Eisner, a military toxin contaminates a town's water supply and causes townspeople to go into violent frenzies and affect their sleep and mental state. In Equilibrium (2002) by Kurt Wimmer, an emotion‐suppressing drug (Prozium) is mandated by the state. Emotional flattening leads to reduced dream activity and disrupted sleep as a side effect of neurochemical compliance. In Inception (2010), directed by Christopher Nolan, participants enter others' dreams through the use of sedative compounds. The film interrogates the structure of dream consciousness and its overlap with real‐world sleep states. In Limitless (2011), a nootropic drug enhances brain function, granting sleepless productivity. Yet, the withdrawal effects and the body's ultimate dependence reflect real‐world issues of stimulant overuse and the unsustainability of ‘sleep‐less’ ambition. In Vanilla Sky (2001) and its Spanish original *Abre los ojos* (1997), cryosleep intersects with lucid dreaming in a tech‐enabled afterlife. While no pharmacological substance is used per se, the company ‘Life Extension’ employs a sophisticated neuro‐technological protocol called ‘Lucid Dream’, a form of synthetic, dream‐like consciousness maintained during cryogenic suspension. This technological ‘sleep’ simulates a perfect mental reality, raising philosophical questions about memory, desire and perception. The series *Altered Carbon* (2018–2020), based on the novel by Richard K. Morgan, introduces a future in which consciousness can be transferred between bodies. Biological sleep becomes optional, and identity is untethered from rest, raising philosophical questions about embodiment and continuity.

Exposure to environmental toxins is clearly linked to sleep alterations. Heavy metals (like lead, cadmium, antimony, etc.), pesticides, and air pollutants have been linked to sleep disorders, potentially through mechanisms such as inflammation, oxidative stress, endocrine disruption, or direct interference with neurotransmitter systems regulating sleep. For example, lead exposure has been linked to altered sleep patterns and increased risk of sleep disorders (see, e.g., Wani et al. 2015). Pollutants in the air can lead to respiratory issues that disrupt sleep, as well as systemic inflammation that can further impair sleep quality (see Azadi et al. 2025). Environmental toxins may increase the risk of central hypersomnia such as narcolepsy (Niede and Benbi 2022).

Viral and bacterial infections provoke inflammation, fever and respiratory symptoms, all of which can affect the ability to fall asleep or maintain restful sleep. Conversely, some antibiotics are known to negatively impact sleep. Respiratory viruses, like the flu or COVID‐19, often lead to sleep disturbances due to symptoms such as coughing, fever and body aches. Additionally, viral infections can directly alter circadian rhythms and sleep architecture (Borrmann et al. 2021; Zhuang et al. 2017).

If there were ever a parasite designed by a malevolent SciFi villain to tamper with human sleep, Trypanosoma brucei would be it. Patients with the fatal ‘sleeping sickness’, caused by this parasite, have disruptions of circadian rhythms, thermoregulation and sleep. In a mouse model with persistent brain infection but cleared acute symptoms, infected animals showed altered sleep patterns and a blunted response to sleep deprivation. These effects appear linked to reduced adenosine signalling, a key pathway in homeostatic sleep regulation (Rijo‐Ferreira et al. 2020). Drugs (for an overview see Spiegelhalder et al. 2025), whether prescribed or recreational, can significantly impact sleep patterns and quality, and physiology. Medications like benzodiazepines and barbiturates are designed to promote sleep but can lead to dependence and impaired sleep quality over time. Caffeine and other stimulants can disrupt sleep, leading to difficulties in falling asleep or staying asleep. Psychedelics and hallucinogens like LSD or psilocybin may alter dream perception and are being explored for their potential to shed light on sleep and consciousness mechanisms. A recent systematic review identified 73 medications linked to conditions such as nightmares, REM sleep behaviour disorder, sleepwalking, restless legs syndrome, periodic limb movements, and bruxism. These effects are thought to involve serotonergic, dopaminergic, orexinergic and GABAergic pathways, with antipsychotics, opioids and other CNS‐active drugs frequently implicated. While rarely mentioned in drug labels, these side effects are increasingly recognised and may be underreported, highlighting the need for further pharmacovigilance and clinical awareness (Dumont et al. 2025).

Using drugs or engineered biological agents to control sleep can lead to issues of autonomy and free will, and so raises fundamental ethical concerns. If society can dictate how and when individuals sleep, it raises concerns about autonomy, mental privacy and free will, and blurs the line between care and coercion. The use of drugs or toxins to alter sleep raises questions about the long‐term health impacts. Chronic use of sleep‐inducing medications is associated with well‐documented risks, including dependence, tolerance, withdrawal syndromes and long‐term disruption of natural sleep architecture. Over time, these alterations can affect cognition, emotional regulation, immune function and even mortality. As such, interventions designed to improve sleep must also be assessed for their long‐term systemic impacts (de Mendonça et al. 2023). If sleep‐enhancing drugs, or microbiome‐based interventions or technologies become available, disparities may arise between those who can afford such treatments and those who cannot. This raises questions of health equity, particularly if cognitive or professional performance becomes tied to access to sleep‐related enhancements. As research progresses, the integration of viruses, bacteria, drugs and toxins in sleep science could lead to fascinating developments. Here are some of these perspectives: (a) Advancements in synthetic biology and biotechnology may soon enable highly targeted interventions, such as engineered microbes or gene therapies that treat sleep disorders more effectively, possibly with fewer side effects than traditional pharmaceuticals. (b) The future may see the creation of safe, effective drugs designed to enhance sleep quality and cognitive function, potentially allowing individuals to achieve optimal mental performance without the drawbacks of current sleep medications. For instance, from nootropics to REM‐boosting compounds, future sleep interventions may aim not just to restore but to optimise memory consolidation, learning and emotional processing. (c) In speculative futures, sleep itself could be weaponised. Sleep could become a target for biological warfare, where engineered agents disrupt populations' sleep patterns, affecting productivity and mental health on a large scale. Disrupting a population's sleep patterns could destabilise entire societies by impairing mental health, cognition and resilience.

Conclusion: The intersection of viruses, bacteria, drugs and toxins with sleep science is a complex and thought‐provoking area, both in scientific research and in science fiction narratives. It opens up both promising and troubling frontiers. These elements raise important questions about human biology, control, autonomy and the ethical implications of manipulating fundamental aspects of our lives. As we explore the boundaries of science, the speculative possibilities presented in literature and film serve to caution and inspire discussions about the future of sleep and consciousness. By pushing the limits of scientific innovation, we must remain vigilant about the ethical, biological and societal consequences of manipulating our most fundamental rhythm: the need to sleep.

## 7 Dreams

Dreams have fascinated humankind for thousands of years. As landmarks, the *Dreambook* by Artemidorus von Daldis (probably dating back 2000 years; here quoting the German edition from 1753), Sigmund Freud's *Interpretation of Dreams* (1900) and more recently experimental dream research (see Pace‐Schott et al. 2003), among many other important contributions, have to be mentioned. One major popular interest was (and probably still is) the interpretation of dreams, with the aim to decipher our strange and vivid nighttime hallucinations, which can oscillate between being rather trivial to depicting the strangest scenarios and stories. It was Freud (1900) who coined the notion of dreams constituting the royal road—via regia—to the unconscious. A major push for dream science came in Aserinsky and Kleitman 1953, when Aserinsky and Kleitman discovered REM sleep, which was assumed to be the physiological basis of our human dreaming experience. This led to a variety of experimental approaches to dream studies in the sleep laboratory, with its heyday in the 1960s–1970s (see, e.g., Arkin et al. 1978: The Mind in Sleep). In the meantime, much of the initial enthusiasm has died down, and dream research at present has shrunken to a much smaller field within the whole sleep research and sleep medicine arena. A major reason for that may have been the fact that in the meantime we know through experimental study that the relationship between the experience of dreaming and sleep state is far from being a simple 1:1 isomorphism with the REM sleep state.

Dreams are very richly represented in Sci‐Fi literature and films. H. P. Lovecraft's Dream Cycle, including *The Dream‐Quest of Unknown Kadath* (1926–1927), uses dreams as a way to travel to otherworldly dimensions. The boundaries between the waking world and the dream world are fluid, and dreamscapes reveal ancient, cosmic horrors that are otherwise inaccessible to human understanding. Jorge Luis Borges (though not strictly Sci‐Fi) often influenced the genre with his short stories about dreams, time and the nature of reality. In stories like *The Circular Ruins* (1940), Borges explores the idea of dreams as creative acts, where a man dreams another person into existence, only to realise he himself is someone else's dream. This existential and recursive treatment of dreams mirrors the type of metaphysical speculation found in Sci‐Fi literature. Frank Herbert's *Dune* (1965) integrates prescient dreams into the spiritual and political intrigue of the story. The protagonist, Paul Atreides, has dreams that predict the future, allowing him to navigate complex political and social dynamics while also exploring the role of prophecy and destiny. Philip K. Dick often uses dreams to question reality and the nature of existence. In ‘Ubik’ (1969) and *Do Androids Dream of Electric Sheep?* (1968), characters experience dreamlike or altered states of consciousness that challenge their perceptions of reality. The dream motif reflects a key concern in his works: how can we trust what we perceive as reality, especially in a world increasingly mediated by technology? Ursula K. Le Guin's *The Lathe of Heaven* (1971) is one of the most famous Sci‐Fi novels focused explicitly on dreams. The protagonist, George Orr, has the ability to alter reality through his dreams, and his psychiatrist tries to harness this power to create a utopia. The book explores the ethical implications of controlling dreams to reshape the world and addresses how unconscious desires might manifest if given the power to rewrite reality itself. In Stanislaw Lem's *The Futurological Congress* (1971), a hallucinatory future reality results from drug‐induced states where people are trapped in dreamlike visions. Lem critiques both utopian aspirations and dystopian fears through surreal, dream‐based experiences where the boundaries between a ‘real’ future and its fantastical representation blur. William Gibson's *Neuromancer* (1984) and the *Sprawl Trilogy* introduce ‘cyberspace’ as a kind of waking dream where the line between virtual and physical reality blurs. Neuromancer's characters frequently traverse a digital landscape where their experiences, though artificial, feel as vivid as dreams, emphasising the malleability of reality in a technologically advanced future. Dreams also serve as a vehicle for horror and existential dread in Sci‐Fi. Stephen King's *Dreamcatcher* (2001) blends alien invasion with dream manipulation. The characters, who share a telepathic bond, experience terrifying dreams linked to an alien presence. King often taps into the idea that the subconscious mind, through dreams, can reveal horrors or allow invasions from the unknown, as in *The Stand* (1978). Alastair Reynolds' *Revelation Space* series explores the idea that dreams, or dream‐like experiences, may be a sign of evolving consciousness. In the far future, humans and post‐humans (beings who have evolved far beyond normal humanity) experience heightened states of consciousness through dreams, often touching upon cosmic awareness or interstellar understanding.

In Solaris (1972), (2002), the film based on the novel by Stanislaw Lem, the characters aboard a space station orbiting a mysterious planet experience vivid dreams and hallucinations that manifest their deepest fears and desires. The boundary between sleep and wakefulness blurs, symbolising the fragility of human consciousness when confronted with an alien intelligence. In A Nightmare on Elm Street (1984), sleep is the very source of horror. The villain, Freddy Krueger, attacks his victims in their dreams, turning the act of sleeping into a death sentence. Here, sleep becomes a symbol of ultimate vulnerability, as characters have no escape once they close their eyes. In The Matrix (1999), the idea that everyday life is an artificial dream‐like simulation is a central theme. While the characters are not technically asleep, their experience within the Matrix mirrors the sense of waking up from a dream, a metaphor for consciousness trapped in a digital reality. In *Minority Report* (2002), based on Philip K. Dick's short story, sleep plays a role in the pre‐crime division's use of precognitive individuals who are kept in a semi‐conscious, dream‐like state to predict future crimes. Here, sleep is used as a tool of control and exploitation. Eternal Sunshine of the Spotless Mind (2004) uses sleep as a framework for memory erasure, with characters undergoing the process while unconscious. Dreams and memories blend together, raising questions about identity, memory and the manipulation of human consciousness. Inception (2010) by Christopher Nolan is a prime example of dreams being manipulated to achieve specific goals. The characters can share dreams and even implant ideas within a person's subconscious, raising questions about the nature of reality and free will. The multiple layers of dreams within dreams create a narrative where the boundaries between wakefulness and dreaming are constantly blurred.

Confronting the possibilities of Sci‐Fi with what has been achieved and is going on in dream research is rather sobering. Actually, probably the first experimental study trying to influence dreams was already published in 1867 by the French noble Hervey de Saint‐Denys. He seems to have experimented with applying sensory stimuli during sleep, sometimes finding that the stimuli were somehow incorporated into dreams reported after awakening. More importantly, he probably was a natural lucid dreamer, writing ‘I dream of being excited sexually by a person who does not excite me in waking life. That this person excites me seems incongruous and I deduce that I must be dreaming. I wonder whether I am really excited or only dreaming that I am. In order to find out, I decide to wake up, and I discover that I am not excited’. We will more deeply explore Lucid Dreaming later on. The experimental manipulation of dreams was a focus of sleep laboratory driven research after the discovery of REM sleep. Scientists studied the impact of states like hunger, deprivation of all kinds or simply applied stimuli like water spray, fragrances etc. to sleepers in the REM sleep state. Largely, it was found that some sleepers did incorporate sensory stimuli, for example, reporting dreaming of rain when they had been sprayed with water and so on. States of deprivation led either to dreams reflecting the situation (continuity) or of the contrary (compensatory). For example, people food deprived for a day either dreamt of a shortage of food or of eating. At present, the most influential theory of what influences dreams is the continuity hypothesis (Schredl 2010), postulating that dream do reflect what drives us, what we need and what holds our present attention and is important for us. Dream interpretation, as important as it was thought to be just a century ago, has lost much of its momentum and does not play an important role in most forms of psychotherapies anymore. A major reason for this may have been the fact that a dream interpretation following scientific principles of reliability, objectivity and validity does not seem to be possible. Instead, dream research turned to dream content analysis, working with more objective, reliable and valid scales to describe dreams, not interpreting them. Most of these scales have been summarised by Winget and Kramer (1979) in their volume ‘The dimensions of dreams’. This type of research did not produce spectacular insights into the human psyche, but led to a catalogue about what we dream, depending on our social status, sex, profession, etc. An interesting clinical application of dream research constitutes the psychotherapeutic treatment of nightmares, which typically occur out of REM sleep. Krakow and Zadra (2006) coined the term ‘Imagery Rehearsal Therapy’ (IRT) for an exposure‐based intervention successfully reducing nightmare frequencies in different clinical populations, among them war veterans and patients with post‐traumatic stress disorder. IRT is a short‐term therapy instructing patients to record their nightmares, reflect them and to re‐write the script in a more harmless way for the afflicted dreamer—the evidence for this approach is substantial (Casement and Swanson 2012).

The phenomenon of Lucid Dreaming (LD) is not seen as controversial anymore (for overview see: Baird et al. 2019; Ableidinger and Holzinger 2023). LD is extremely popularised, promising everybody the capability to control and improve his or her dreams, with hundreds or even thousands of web pages and internet videos promising to teach you how to become a lucid dreamer in just a few steps. LD is defined as the capability to regain normal consciousness during dreaming, that is, an LD realises that he or she is dreaming when in a dream state. In a next step, the LD sets out to modify the ongoing dream by certain techniques, with the aim of tailoring it to the needs of the dreamer. In a scientific vein, LD seems to hold promise for consciousness research or even nightmare therapy. Furthermore, in one relatively small study, a group of individuals with insomnia were taught to lucid dream and showed it improved their symptoms as well as resulting in reductions in anxiety and depression (Ellis et al. 2021).

Conclusion: Given our overview here, most dream‐related concepts of Sci‐Fi are not reflected as research topics, probably because they are too far‐fetched or seem improbable or impossible to realise. To put it simply: at present we cannot directly manipulate the dreams of others or write their dream stories for them or even invade their dreams. That said, they may be used therapeutically in a self‐help framework, although more research is needed.

## 8 Conclusion

After having reviewed several areas from the viewpoints of Sci‐Fi and sleep science, we have to admit that our overview is far from being exhaustive or complete. However, we hope the chosen concepts we investigated reflect the main areas of interest in Sci‐Fi, with some overlap between the different fields. We would now like to judge the scientific feasibility of the several areas. Hypnopaedagogics, at least in our opinion, with its claim of effortless learning during sleep, is likely not going to materialise. Space hibernation and cryosleep are matters of serious scientific study—it seems feasible that this approach will serve medical research in the near future, for example, for the preservation of organs. Whether space hibernation of humans for years or even longer will ever happen is more unclear. BMIs in general and electrosleep approaches already exist and some promise is seen from our side here concerning new ways of influencing sleep. Genetic engineering of sleep is already taking place successfully in experimental animal models—the question is whether these technologies can be translated without adverse consequences to humans. All kinds of substances and drugs have been studied by investigators in the sleep lab in animals and humans, and significant progress has been made, for example, for the treatment of narcolepsy. It will have to be seen whether in the near future a drug will be developed inducing natural sleep having no adverse effects at all, of which we all dream in the field of sleep medicine. Dreaming as such seems rather immune to manipulation by others, and we do hope that this very intimate experience will be cautionary to technological efforts to optimise dreaming.

As mentioned at several points in our manuscript, societal and ethical issues have to be considered when analysing the Sci‐Fi work and possible scientific realisations. Much of the Sci‐Fi literature and films explicitly use the fictional possibilities as a framework for exactly addressing these problems and, no wonder, are dystopic in nature. Thus, scientific work should adhere to the principles outlined by Ethical guidelines.

It is difficult to say whether Sci‐Fi instigated scientific studies in a given field or whether scientific ideas were taken up by Sci‐Fi writers or filmmakers. In many cases, at least to us, it seems that Sci‐Fi followed science, adding the fictional elements to what was scientifically en vogue. Needless to say, we are not aware of any Sci‐Fi work going beyond using fictional elements by describing how they work exactly.

Interestingly, when having a closer look at Sci‐Fi, to us it becomes clear that works of Sci‐Fi are much more about psychology, society and the human experience than about technology. A shift of perspective from the ‘normal’ world to a Sci‐Fi world is frequently used to explore human psychology under different circumstances.

## Author Contributions

**Dieter Riemann:** conceptualization, writing – original draft, writing – review and editing, supervision, formal analysis. **Christoph Nissen:** conceptualization, writing – original draft, writing – review and editing, supervision. **Pierre A. Geoffroy:** conceptualization, writing – original draft, writing – review and editing. **Bernd Feige:** conceptualization, writing – original draft, writing – review and editing. **Jason Ellis:** conceptualization, writing – original draft, writing – review and editing.

## Conflicts of Interest

The authors declare no conflicts of interest.

## Data Availability

Data sharing not applicable to this article as no datasets were generated or analysed during the current study.
